# Self-organized criticality in a model for developing neural networks

**DOI:** 10.1186/1471-2202-12-S1-P221

**Published:** 2011-07-18

**Authors:** Benjamin van den Akker, Borja Ibarz, Raoul-Martin Memmesheimer

**Affiliations:** 1 Department of Neuroinformatics, Radboud University, Nijmegen, 6525AJ, Netherlands; 2 Center for Neural Science, New York University, New York, NY 10003, USA

## 

Recent experiments have observed a dynamical state characterized by so-called neural avalanches in different neural systems, such as networks of cultured neurons [1], the developing retina [2] and the neocortex in vivo [3]. Neural avalanches are bursts of activity that have power-law size distribution, which suggests that the system has assumed a critical state. To investigate how such a state might develop, we study neural network growth models that were proposed on the basis of neurobiological experiments [4,5]. In these models, the spiking activity of a neuron governs the outgrowth of its processes and the spatial overlap between neuronal processes determines the coupling strengths. We show analytically that an appropriately modified version of these models self-organizes into a state where it generates critical spiking activity and neural avalanches, i.e. the network grows into criticality. The conditions under which this happens are studied analytically and numerically. We complement our findings by investigating the structural and dynamical properties of the network, such as the lengths of neural protrusions in different one- and two-dimensional spatial arrangements and the temporal correlations between avalanche shapes and sizes, during development and in the critical state.
